# The Book World of Medicine and Science

**Published:** 1893-09-09

**Authors:** 


### THE BOOK WORLD OF MEDICINE AND SCIENCE.

### BOOK REVIEWS.

Some Lectures by the Late Sir George Paget, K.C.B., M.D., F.R.S., Regius Professor of Physic in the University of Cambridge. Edited from MSS., with a memoir, by Charles E. Paget, Vice-President of the North-Western Branch of the Incorporated Society of Medical Officers of Health, Member of Council of the Epidemiological Society of London, and Medical Officer of Health for the Borough of Salford. (Cambridge: Macmillan and Bowes. 1893.)

The charm, and one might truly say the value, of this book lies not in the lectures, but in the memoir that precedes them. Not that the lectures themselves are worthless, or require the excuse of filial piety for their publication. They are, indeed, models to many writers in moderation of tone, simplicity of expression, carefulness of observation, and freedom from vanity and dogmatism. But we have many writers on typhoid fever and the injurious effects of alcohol; we could better do without the subject matter of the lectures than the moral lessons we may derive from the tone in which they are written. That same tone breathes even more unmistakably in the memoir, and in these days when some of our prominent medical men have injured their own repute and that of their profession by self-seeking and self-advertisement, a life like that of Sir George Paget comes before us with double emphasis.

It is rather curious to find that circumstances rather than his own will first made him a doctor. He was led to medicine through success in mathematics. The mathematics were, in the first instance at least, self-acquired at the Charterhouse School, where he had for schoolfellows Edmund Law Lushington, afterwards Professor of Greek in Glasgow University, and that brother-in-law of Tennyson's whom the Laureate described as wearing "all that load of learning lightly like a flower," with George Richard Venables, and with William Makepeace Thackeray. At the Charterhouse in these days a knowledge of the classics was deemed to be all that was necessary to a liberal education. But a London banker, whose son was at the school, was shocked to find that his expensively-educated boy could not add up a sum in compound addition. He remonstrated with Dr. Russell, the head master, who allowed himself to be persuaded to engage one man to teach arithmetic and mathematics to four hundred boys. Alas! poor teacher! In this class George Paget came out first, he having before this, for his own good pleasure, studied the first book of Euclid. Still devoting himself to mathematics, Paget went to Cambridge, where in the mathematical tripos he came out eighth wrangler. His position in the honours list was rewarded by a fellowship of his college (Gonville and Caius), the terms of which demanded that the holder of it should be a native of Norfolk, and a member of the medical profession.

The birth status Paget possessed, having been born at Great Yarmouth in 1809, and he set himself to acquire the other by commencing a course of medical studies at Cambridge, completing them at St. Bartholomew's and in Paris. He took the degree of M.B. in 1833, and that of M.D. in 1838. The following year he became a Fellow of the Royal College of Surgeons, and was appointed physician to Adden

**THE BOOK WORLD OF MEDICINE AND SCIENCE**—*continued.*

brooke's Hospital. Then followed many busy years, in which neither the demands of a wide consulting practice nor recurring attacks of rheumatic fever prevented him from interesting himself in the affairs of the University. Among other things, he strongly urged upon the University authorities the encouragement of the study of natural science, and giving to success in the natural science tripos, which at last, in 1851, he saw established, some of the distinctions and rewards which had till now been kept exclusively for mathematics and classics. This was the last service he was able to do directly for the University for some time; for in the same year (1851) he married, and under the existing statutes thereby forfeited his fellowship. But the esteem in which he was held was marked 30 years after, when in 1881, under the new University statutes, his old college elected him a Professorial Fellow. He had, however, been a member of the Council of the Senate since 1856, and had been elected and re-elected every four years Linacre Lecturer in Medicine at St. John's College, until he was appointed Regius Professor of Physic in the University.

His history shows, as his son says, "personal energy, hearty adoption of his profession, the esteem of his fellows, and an upright and genuine regard for the terms on which he held his fellowship." His own feeling for his profession may be inferred from a passage in the address he delivered in 1864, when he was President of the British Medical Association, which that year met at Cambridge. He especially urged upon his listeners the necessity of keeping up a high standard of scientific attainment. "Every ignorant man admitted into our profession," he said, "has an injurious influence on the estimation in which the entire body is held. His demerits have a tendency to lower us throughout the circle in which he is known. The want of confidence in him, the want of respect for him, begets distrust and disrespect for the profession in general." One feels glad to think that this *preux chevalier* of medicine received the honour of knight hood, even in his old age, in 1885. His published works were few, but thoughtful and accurate, and he was admittedly one of the most careful of observers in clinical medicine. His students and his patients received the benefit of his studies, rather than the public at large; but his life, with its high ideal, was perhaps his greatest contribution to his profession, and it is one that merits the consideration of all who aspire to follow the hard but honourable profession he adorned.

The New Priesthood. By Ouida. (London: E. W. Allen, 4, Ave Maria Lane, E.C.)

The same lavish and emotional style, the same exuberance of epithet, the same indifference to vulgar accuracy, which marked the Ouida of our youth, when she described fascinating *demi-mondaines* who sprinkled their hair with gold-dust and girdled their waists with ropes of historic pearls, or guardsmen, effeminate yet heroic, whose barrack-rooms resembled in the luxury of their appointments "the boudoir of a young duchess," and who kept necklaces of priceless mosaics lying on their breakfast-tables for presentation to any chance visitor of the necklace-loving sex who might drop in—the characteristics which made "Puck," and "Pascarel," and "Folle-Farine," and a dozen other works successful, fascinating, and unreal, reappear in the Ouida, who in her maturer years has become the champion of the animals sacrificed at the shrine of physiology. No one can

THE BOOK WORLD OF MEDICINE AND SCIENCE—*continued.*

question the sincerity and depth of Miss de la Ramée's feelings. They are simple and womanly, far more simple than her sentences, and as womanly as her reasonings. She loves animals, she hates to think of their being tortured, and she says harsh things of the torturers. She attacks vivisectors, but practically she leaves untouched the whole question of vivisection. And to what do even her attacks on the physiologists amount? This is the style of them:—"Cornelius Herz was, and is, a prominent member of the (physiological) priesthood; his discoveries on the nature and functions of the spine have been vaunted and glorified to us by scientists for years. He received, ostensibly in return for his physiological and electrical work, the Grand Cross of Legion d'Honneur, and he would have certainly, until the last few months or so, ranked among Mr. Hart's men of undoubted honour and honesty. What is his moral character now proven to be? How many like him may be living undiscovered among the scientific tyrants into whose hands the docile world is invited to deliver itself, blindfolded and unquestioning?" This tirade, translated into the vulgar tongue, means that because Cornelius Herz is implicated in the Panama scandals his work in the physiological laboratory is to be held of no account. Then in strict logic we must assume also that because Ferdinand de Lesseps embezzled public money the Suez Canal is useless, and it is our moral duty to go to India round the stormy Cape. The one piece of reasoning is as good as the other. That a man has committed one sin does not prove that he has broken all the Decalogue. There is a natural selection, a taste, even in vices. Has not Ouida herself depicted for us a score of times the man whose life seems, to us of the narrow-minded middle class, to be a record of selfish and meaningless debauchery, who yet can fight like a hero and die like a martyr? Dr. Herz's investigations on the functions of the spine must be judged by one tribunal, his relations to Reinach by another; we must not confound two things which have no necessary connection. His physiological discoveries shall not condone his thefts, neither let his thefts condemn his physiology.

It is not only for the lower animals that Ouida feels; her sympathies are as deeply aroused for "the poor victim or the hospitals, helpless as the dog tied down to the torture-trough, unable to resist, ignorant of what is done, offered as a spectacle or as a subject to the students, lying prone and motionless on his bed of pain, angrily silenced if he dare protest, experimented on against his will, or without his knowledge; mere 'material' as he is, to be studied, pulled about, probed, tormented, lacerated, until he can bear no more, and faints and dies, and is carted away a useless mass of mutilated tissue, like the used-up dog flung out of the hospitallaboratory with excoriated nerves and opened breast."

This is a simple and concise summary of hospital practice! If Ouida's ideas were carried out no new operation would be performed, no new treatment essayed; our students would go into the world without having seen anything of the diseases they profess to cure. The strange thing is that such of these victims as do not "faint and die" are so willing to come back to the hospital when they fall ill again. They must, these poor human brutes, be under the delusion that the hospital treatment was not unkindly meant, and even did some good.

Of course no work, however short, of this talented writer would be complete without a fling at her own sex—"woman

THE BOOK WORLD OF MEDICINE AND SCIENCE—*continued.*

who, when once she is cruel, is tenfold more cruel than man, and when she is pitiless is a hundredfold more pitiless than he." The medical woman finds no favour in Ouida's eyes. Hear how the whole question of the lady doctor is summed up: "Women are received in medical schools and vivisecting chambers, and help to increase the multitude which intends to live by the scalpel and the hypodermic syringe. There will be, in the future, between the sexes, fierce, brutal, pitiless competition for place and for practice. Into the hands of these men and women have been put the power and the means of communicating the deadliest maladies without detection. Under pretext of healing one disease they can communicate a worse."

This assumption, that in educating doctors of either sex we are simply developing potential murderers is sufficiently startling, and Ouida thinks it impressive enough in itself to do without any support from facts. Indeed, facts are beneath her notice. Only on one point does she speak from proved incidents. This is the development of a nervous, hysterical fear of infectious disease which has sprung up among the ignorant or half-educated from hearing, without wholly understanding, about the sources of infection. It is likely enough that this has led to acts of inconsiderate panic and cruelty, such as she quotes as having occurred in an emigrant train in Canada, when a case of small-pox was discovered, and the inhabitants of the nearest town refused to let the patient be received there. But an exactly parallel case occurred a year ago in the island of Mull, where not a cottager would give the shelter of even an outhouse to a pedlar who was supposed to be suffering from typhus fever, and there assuredly the inhumanity arose from no knowledge of sanitation or bacteriology spread by scientists. But people feared certain diseases long before bacteria were ever spoken of. It does not take an intellect rendered more acute, a nervous system made more sensitive and timorous, by scientific study, to perceive that those who associate with small-pox and typhus are likely to contract these diseases; and to receive and tend those suffering from them requires a higher morality than the natural man, scientific or unscientific, possesses. Charity does not, as Ouida seems to think, spring exclusively from ignorance. Knowledge does not destroy courage, and one could bring many an instance from the histories of those degraded beings who live by the scalpel and the hypodermic syringe as worthy of record as the famous tale of St. Francis kissing the leper, and marked by more profitable results to the leper.

Might we venture to say that these results perhaps spring in some degree from the knowledge gained by vivisection? It is not necessary to enter upon the discussion of that question, for Ouida, in "The New Priesthood," does not really touch upon it. Vivisection must be justified or condemned on scientific grounds, and Ouida is not scientific. She speaks, now like a poet, now like a scold, but never, by any chance, like a scientist. To which accusation she may, very probably, respond by a fervent *laus Deo*. For she has no respect for science. "The fanaticism of science," she says, "is as exactly like the fanaticism of religion as one pea is like another. Each has the same blindness, the same egotism, the same pitilessness, the same arrogance, the same hypocrisy." We are forced to add that in all these points they resemble a third creature of the same breed—the fanaticism of ignorance.

